# Differentiation of beta thalassemia trait from iron deficiency anemia by hematological indices

**DOI:** 10.12669/pjms.333.12098

**Published:** 2017

**Authors:** Tahir Jameel, Mukhtiar Baig, Ijaz Ahmed, Muhammad Barakat Hussain, Motlag bin Doghaim Alkhamaly

**Affiliations:** 1 Prof. Tahir Jameel, FCPS. Professor of Clinical Hematology, Faculty of Medicine, Rabigh, King Abdul-Aziz University, Jeddah, Saudi Arabia; 2 Prof. Mukhtiar Baig, PhD. Professor of Clinical Biochemistry, Faculty of Medicine, Rabigh, King Abdul-Aziz University, Jeddah, Saudi Arabia; 3 Dr. Ijaz Ahmed, MPhil. Chief Pathologist, Department of Pathology, King Khalid General Hospital, Hafar Al Batin, Saudi Arabia; 4 Dr. Muhammad Barakat Hussain, PhD. Assistant Professor of Microbiology, Faculty of Medicine, Rabigh, King Abdul-Aziz University, Jeddah, Saudi Arabia; 5 Dr. Motlag bin Doghaim Alkhamaly, MBA Head of Department, Department of Pathology, King Khalid General Hospital, Hafar Al Batin, Saudi Arabia

**Keywords:** β-Thalassemia trait, Iron deficiency anemia, Premarital screening, Red Cell Distribution Width Index (RDWI)

## Abstract

**Objective:**

We aimed at finding out reliable parameter in the differentiation of iron deficiency anemia (IDA) and beta-thalassemia trait (β-TT) in the adult population subjected to Saudi Arabian Premarital Screening Program.

**Methods:**

A total of 620 adults (age range 21-36 years) reported during February 2012 to November 2012. Tests for serum iron and ferritin were carried out in individuals showing low hemoglobin (Hb). All the selected subjects’ samples were subjected to blood morphology, comparison of MCV, RBC count. Red Cell Distribution Width (RDW) was noted from the Coulter Report whereas Red Cell Distribution Width Index (RDWI) value was calculated for all the samples.

**Results:**

A total of one hundred &thirty-five individuals with hypochromic microcytic anemia having normal hemoglobin F and hemoglobin A2 < 3.2% were inducted in the study. Ninety-three were diagnosed having IDA, whereas thirty-two were having βTT. Ten individuals revealed other causes of anemia. The RBC count was higher, and MCV was much lower in βTT as compared to IDA. Both groups were subjected to RDW and RDWI, however, RDWI which showed better sensitivity and specificity for βTT.

**Conclusion:**

RDWI is a reliable and useful index for differentiation among IDA and βTT, as compared to RDW.

## INTRODUCTION

Beta Thalassemia is widely prevalent in Eastern and Southwestern provinces of Saudi Arabia, where the incidence of consanguineous marriages is more than 50%.^1^ A recent study on genetic pattern mentioned that autosomal recessive genetic disorders are highly common and prevalent in Saudi population in specific areas.^2^

Since the start of premarital screening in 2004, the incidence of Thalassemia has dropped dramatically throughout the Kingdom of Saudi Arabia.^3^ The professional staff involved in the decision making regarding the presence of thalassemia and other hemoglobinopathies in the under-screening individuals carry a huge responsibility. At times one faces problem in individuals presenting with low Hb and MCV i.e. hypochromic microcytic picture, revealing HBA_2_ less than the cutoff limit of >3.2% and having low serum iron and ferritin.^4^

Microcytosis and hypochromia are the common presentations of both the disease processes of iron deficiency anemia (IDA) and beta-thalassemia trait (βTT). The morphological findings in both the IDA and βTT are at times so close that it is really difficult to differentiate one from the other.^5^ Differentiation between βTT and IDA can be carried out effectively by involving the battery of tests including serum ferritin, serum iron and HbA_2_ level estimation.^6^ One must be vigilant to rule out relatively less common causes of this presentation, such as sideroblastic anemia, chronic disorder, lead poisoning, and others.

The differentiation between IDA and βTT is important because of two main reasons, first, because Hb won’t improve in βTT if it is misdiagnosed as IDA and unnecessary iron being prescribed by the attending physician.^7^ The second grave reason is that misdiagnosed βTT as IDA may get married to a βTT, resulting in homozygous or thalassemia major in the offspring.^8^

Ideally, one needs a battery of tests including detailed peripheral blood picture, Hb A_2_ estimation, serumiron, TotalIron Binding Capacity (TIBC), serum ferritin and transferrin saturation to differentiate IDA from βTT clearly.^9^ But all these tests are either not available in all clinical set ups, or these are relatively time-consuming and expensive techniques. A number of studies have revealed that derived red cell indices including Red Cell Distribution Width (RDW) can be very helpful in differentiation of anisocytosis caused by IDA or βTT and a recently added Red Cell Distribution Width Index (RDWI) provide valuable help to the attending physician.^7^,^9^,^10^ RDWI is more advantageous as all the discriminating factors including RBC count, MCV and RDW are incorporated in its formula.^11^

We focused on individuals who were found to be anemic but were either having features of IDA or βTT. Thalassemia minor or trait is essentially asymptomatic, but the only finding is mild anemia unresponsive to medicine. RBC morphology resembles strongly to iron deficiency anemia with few differences i.e. rarely seen nucleated RBCS, normal electrophoretic mobility and alkali resistance in Hb.^6^ Fetal Hb is not increased& the characteristic, and diagnostic rise in HbA2 are often not seen in coexisting iron deficiency.^12^

Derived indices like an index of RDW can be calculated using the automated blood cell counters for differentiation between IDA and βTT. The RDW measures the average RBC size variation, calculated by the RBCs histogram and is calculated as a standard statistical value, the coefficient of variation of the volume distribution. Many studies have revealed that RDW is the first index to become abnormal in iron deficiency.^13^-^15^ A rather improvised index, RDWI has proven to be a reliable discrimination index in the differentiation of βTT and IDA.^11^ RDWI can be easily calculated as (MCV x RDW /RBC). Our study aimed at diagnostic comparison of both the RDWI and RDW in the differentiation of IDA and βTT.

## METHODS

This prospective cross-sectional study was conducted from February 2012 to November 2012 at the Department of Pathology at King Fahd General Hospital – Hafar Al Batin, Saudi Arabia. The hospital administration granted permission for the study and the study was carried out according to Helsinki Declaration of Human Rights and informed consent was taken from all the subjects. Healthy couples, who were intending to get married attended marriage consultation center located in the hospital and underwent mandatory tests.

Venous blood was taken into an EDTA tube, the CBC and RBC indices were measured by Coulter Automated Cell Counter (LH500) on the same day of collection. The Hb electrophoresis was done on cellulose acetate.

Two additional tests of serum iron and ferritin were carried out in individuals having a hypochromic microcytic picture (hemoglobin < 8 gram/dl and MCV <80fl). All the selected samples were subjected to blood morphology, comparison of MCV, RBC count, RDW, and RDWI. Cases were diagnosed as IDA and βTTon the basis of two standard tests, Hb electrophoresis and serum iron plus ferritin estimation. Patients with HbA_2_ more than 3.2% were identified as βTT cases and patients with serum ferritin less than 12 ng/ml were identified as IDA cases.^12^ CBC reports including all the indices plus RDW were obtained by the coulter automated analyzer LH500 in all the candidates. The value of RDWI was calculated from parameters provided by the automated analyzer. The cutoff values of RDW & RDWI for differentiation are shown in Table-II.

**Table-I:** Complete blood count (CBC) parameters in both the iron deficiency anemia (IDA) and beta-thalassemia trait (βTT).

*CBC parameters*	*IDA*		*βTT*		

	*Range*	*Mean ±SD*	*Range*	*Mean ±SD*	*p-value*
Hemoglobin (g/dl)	6.8-7.9	7.2 ± 0.7	6.5-7.7	7.3 ± 0.8	0.529
RBC (10^12^/l)	3.65-4.9	4.1± 0.6	5.5-6.2	5.81±2.2	<0.001
MCV (fl)	62.3-79.4	73.5± 1.3	51.1-57.9	53.2±2.5	<0.001
MCH	18.2-28	24.2 ±2.0	14.7-25.5	18.8±1.8	<0.001
MCHC	23.8-39.7	31.6 ±2.2	26.3-38.9	35.2±3.0	<0.001
Ferritin (μg/l)	2.6-9.7	5.02 ±2.1	11.5-88.3	40.3±3.8	<0.001
RDW	13.2-13.9	13.6 ±0.5	10.5-19.6	16.5±1.8	<0.001
RDWI	19.1-21.1	20.2 ±0.6	9.7-18.6	12.5±2.5	<0.001

**Table-II:** Cutoff values of RDW& RDWI for iron deficiency anemia (IDA) and beta-thalassemia trait (βTT).

*Red cell indices*	*β-TT*	*IDA*
RDW (%)	<14	>14
RDWI	<220	>220

RDW=Red Cell Distribution Width, RDWI=Red Cell Distribution Width Index.

Analysis of the data was carried out on SPSS 16. The frequency and percentages were calculated for qualitative data and mean and standard deviation for quantitative data. The student t-test was used to investigate the difference between CBC parameters of IDA and βTT, and P values <0.05 was considered to be as significant.

## RESULTS

Out of 620 individuals undergoing premarital screening, 135 revealed low Hb and low MCV (Hb < 9 gram/dl & MCV <80fl) and Hb F was undetectable in their blood. These (38 males & 97 females) individuals were enrolledfor the study. Their age ranged from 21 to 36 years with the mean of 24 years ±1.5. According to the criteria mentioned in subjects and methods, ninety-three individuals (20 males and 73 females) were diagnosed having IDA, whereas thirty-two of them (15 males and 17 females) as having βTT. Ten individuals revealed the presence of other causes such as chronic disease and sideroblastic anemia.

The Hb characteristics of both the groups i.e. IDA and βTT are shown in Table-I. The RBC count was found to be higher in patient of βTT (6.8-7.7 x10^12^ /l with the mean of 7.3 x10^12^ /l ±1.12) as compared to IDA patients in which it ranged from 3.6-4.9 x10^12^ /l. The range of MCV in βTT was in the range of 51.1-57.9 fL with a mean value of 53.2fL ±0.53, the corresponding values for IDA were 62.3 – 79.4 fl, with the mean value of 73.5 79 fl±0.85.

MCH and MCHC values did not show much difference among both the groups. Serum Ferritin was remarkably low in patients diagnosed as IDA (2.6-9.7 with the mean of 5.02 whereas its levels were on the higher side in βTT patients. Though in some the patients having hemoglobin A2 above the cutoff limits, the serum Ferritin levels were below the cutoff limit of 15µg/l, indicating the coexistence of IDA and βTT. Table-I. Significant differences of Hb level and MCV, found in between βTT and IDA. Another important RBC parameter for detection of IDA and βTT is RDW (Table-II).

In the present study, the mean RDW was found 16.4% in case of βTT and 16.9% in case of IDA. The results were not statistically significant (p=0.269) whereas the derived index i.e. RDWI showed better discriminative effect between βTT and IDA, as this index had both sensitivity and specificity more than 85% in detection of βTT and IDA. Sensitivity and specificity of RDWI for detection of βTTwas found 94.0% and 88.0%. Again for IDA, sensitivity and specificity were found 88.0% and 86.0% respectively. Youden’s index (YI) takes into account both sensitivity and specificity and gives an appropriate measure of the validity of a particular technique. YI of RDWI was found 83.0, which could be a reliable discriminator between βTT and IDA (Table-III). Though several indexes have been mentioned for the purpose of discrimination between βTT and IDA, in the present study we tried to evaluate a simple, effective and user-friendly index which doesn’t require mathematical calculations.

**Table-III:** Sensitivity, specificity, positive, negative predictive value and Youden’s index (YI) of five parameters in patients with iron deficiency anemia (IDA) and beta-thalassemia trait (βTT).

*Parameters & diagnosis*		*Sensitivity (%)*	*Specificity (%)*	*Positive predictive value (%)*	*Negative Predictive value (%)*	*Youden’s Index*
RBC count	IDA	86	90	73	80	74
	βTT	88	85	88	93	
MCV	IDA	78	83	88	85	66
	βTT	85	80	85	89	
RDW	IDA	80	86	88	65	64
	βTT	84	78	90	76	
RDWI	IDA	88	86	91	88	83
	βTT	94	88	99	97	

## DISCUSSION

The requirement of simple distinguishing parameters between IDA and βTT in a patient presenting with hypochromic microcytic is needed since long as several studies have pointed out the direct effect of coexisting IDA on HbA2 synthesis resulting in confusing levels of HbA2 in βTT and the MCV in such patients would not improve on continued iron therapy.^13^

The differentiation between βTT and IDA, requires Hb A2 estimation by Hb electrophoresis, examination of a peripheral blood film, serum ferritin, iron, TIBC, and transferrin saturation. But being relatively expensive and time-consuming, it is preferred to rely on simple and already available information. Red cell distribution width (RDW) is provided in CBCs by the automated analyzers and can be utilized in the associationwith a derived value RDWI to distinguish IDA and βTT.^15^

RDW denotes anisocytosis. Its value is increased in IDA, and it is near normal or mildly increased in βTT. Although RDW is a valued discrimination index for differentiating βTT and IDA,^16^ our results found that RDW is almost equally elevated in both βTT and IDA and YI was found 2.3, which would not be a good discriminator of βTT and IDA. Similar findings also reported by other studies.^15^,^17^

RDW has been known as a valuable differentiation index against βTT and IDA but in our patients, its results were not conclusive. Though, its values were raised in βTT.^18^ Our results found that RDW is more or less equally elevated in βTT and IDA. The mean values of RDW, found in IDA and βTT were 16.9 (SD ±2.9) and 16.4 (SD ±2.5) respectively. The sensitivity, specificity of RDW in the detection of βTT didn’t prove to be significant whereas RDWI came out as good discriminator between βTT and IDA, its sensitivity and specificity were more than 80% in detection of βTT and IDA. The sensitivity and specificity of RDWI in the detection of βTT were found 80.7% and 84.7%, respectively and the sensitivity and specificity for the detection of IDA were 84.7% and 80.7%, respectively. These results are consistent with the findings of other relevant studies.^19^,^20^

In our study, the sensitivity of 89% and specificity of 94% were observed for RDWI. This isin accordance with the observations made by quite a few studies.^18^,^20^,^21^ In the present study, the highest YI was obtained for RDWI (83). The YI takes into account both sensitivity and specificity and gives an appropriate measure of the validity of technique.^22^ In the present study, YI evaluated the discriminating function of the red cell indices and their derived formulae. The discriminating function of the various indices is dependent on the age of a patient. It has been observed that the most accurate discriminant index for the patients younger than 10 years is total RBC count and for those older than ten years are TRBC and RDWI.^23^,^24^ In a recent study another discriminating index, Matos & Carvalho Index has been mentioned as a very effective discriminating factor in the differentiation of IDA and βTT. It can be calculated by the formula MCI= (1.91xRBC)+(0.44xMCHC)^25^

### Limitations of the study

The limitations of the present study were small sample size, and all red cell indices were not analyzed. There is need for further studies evaluating the discriminating function of all possible red cell indices published in the literature so far, but the present study provides us an opportunity to use simpler methods for differentiation between two confusing states.

## CONCLUSION

RDWI appears to be a reliable and useful index for initial screening of microcytic hypochromic anemia and is better than RDW in differentiating IDA from βTT.

### Author’s contribution

***TJ:*** Conceived the idea, designed the study & edited the Manuscript.

***IA:*** Collected data, reviewed manuscript & critical analysis.

***MB:*** Did statistical analysis, helped in manuscript writing.

***MBH:*** Helped in manuscript writing and scrutinized the data.

***MDA:*** Helped in data collection, and critically analyzed and approved the manuscript.
